# Evaluation of *Corcyra cephalonica* as an Alternative Host for *Meteorus pulchricornis*

**DOI:** 10.3390/insects17050518

**Published:** 2026-05-19

**Authors:** Yan Yan, Xingyu Lin, Rui Shi, Pingli Shi, Xiqian Ye, Xuexin Chen, Aimin Shi, Pu Tang

**Affiliations:** 1 State Key Laboratory of Rice Biology and Breeding, Zhejiang Provincial Key Laboratory of Biology and Ecological Regulation of Crop Pathogens and Insects, Ministry of Agriculture Key Lab of Molecular Biology of Crop Pathogens and Insects, Zhejiang University, Hangzhou 310058, China; yan.23@intl.zju.edu.cn (Y.Y.);; 2 College of Life Sciences, Capital Normal University, Beijing 100048, China; 3 Yunnan Tuidongzhe Biotechnology Co., Ltd., Kunming 650201, China

**Keywords:** *Meteorus pulchricornis*, *Corcyra cephalonica*, alternative host, parasitism, cocooning rate, eclosion rate

## Abstract

*Meteorus pulchricornis* is an important larval parasitoid of lepidopteran pests and has been widely considered for use in biological control programs. Identifying a promising candidate for a factitious host is essential for its efficient rearing. Currently, *Mythimna separata* is the main host for mass rearing of *M. pulchricornis*, but this system faces problems such as high production cost, larval cannibalism, and low rearing efficiency. In this study, we evaluated the suitability of *Corcyra cephalonica* as a candidate factitious host for *M. pulchricornis*, using *M. separata* as a reference host. Host suitability was assessed through parasitism rate, cocooning rate, and eclosion rate under different host body lengths and parasitoid-to-host ratios. In addition, offspring quality was evaluated by measuring adult body length, total egg number, and subsequent parasitism, cocooning, and eclosion performance. Parasitism, cocooning, and eclosion rates did not differ significantly among late instar *C. cephalonica* larvae with body lengths of approximately 10–15 mm. The parasitoid-to-host ratio had a pronounced effect on performance. Parasitism and cocooning rates were highest at a ratio of 1:5 and declined with increasing host density. Offspring derived from *C. cephalonica* showed comparable parasitism, cocooning, and eclosion rates to those derived from *M. separata*, although adult body length and total egg number were significantly lower. Overall, our results provide preliminary evidence that *C. cephalonica* can support the development and reproductive activity of *M. pulchricornis*. However, the reduced body size and egg load indicate that further optimization is required before this host can be considered equivalent to the reference host. These findings suggest that *C. cephalonica* is a promising candidate for a factitious host and may warrant further investigation for the development of rearing protocols.

## Simple Summary

*Meteorus pulchricornis* is an important larval parasitoid of many lepidopteran pests and has great potential for use in biological control. Efficient rearing of this parasitoid depends on the availability of a suitable factitious host. In this study, we evaluated *Corcyra cephalonica* as a candidate factitious host for *M. pulchricornis*, with *Mythimna separata* used as a reference host. We tested the effects of host body length, parasitoid-to-host ratio on parasitism, cocooning, and eclosion performance, and further assessed offspring quality by measuring adult body length, egg number, and subsequent parasitism performance. The results showed that late instars of *C. cephalonica* were all suitable for parasitism, with no significant differences in parasitism, cocooning, or eclosion rates among the fifth, sixth, and seventh instars. Parasitoid performance was affected by parasitoid-to-host ratio, and parasitism, cocooning, and eclosion rates were generally highest at a ratio of 1:5 and declined at higher host densities. Offspring derived from *C. cephalonica* showed comparable parasitism, cocooning, and eclosion rates to those derived from *M. separata*, although adult body length and egg number were lower. These findings suggest that *C. cephalonica* shows as a candidate factitious host for *M. pulchricornis* under laboratory conditions and provide a foundation for further optimization studies.

## 1. Introduction

The increasing demand for environmentally sustainable pest management has renewed interest in biological control strategies that reduce reliance on chemical insecticides [1,2]. Among these strategies, the large-scale use of parasitoid wasps is often constrained not by their effectiveness in the field, but by limitations in mass-rearing systems [3,4,5]. In particular, the selection of suitable factitious or alternative hosts plays a critical role in determining production efficiency, cost, and long-term stability of parasitoid colonies [6,7,8].

*Meteorus pulchricornis* (Wesmael) (Hymenoptera: Braconidae) is a solitary koinobiont endoparasitoid with a broad host range across several families of Lepidoptera. Because of its ecological safety, adaptability, and ability to suppress multiple pest species, *M. pulchricornis* has been regarded as a valuable natural enemy in integrated pest management programs [9,10,11,12]. The species is widely distributed throughout the Palearctic region, including East Asia and Europe [10]. Both bisexual and thelytokous populations have been reported, with thelytoky predominating in several East Asian populations and enabling stable population maintenance without mating [13,14,15,16]. Since its early introduction into North America for the control of *Lymantria dispar*, *M. pulchricornis* has been evaluated against a range of economically important noctuid and pyralid pests, including species of *Spodoptera*, *Mythimna*, and *Helicoverpa* [10,17,18,19,20]. However, translating these favorable biological traits into future scaling applications depends critically on the feasibility of efficient and economical rearing systems [21].

Despite these advantages, the practical use of *M. pulchricornis* in biological control remains constrained by difficulties in mass production [5,22]. Current rearing systems primarily rely on noctuid larvae such as *Mythimna separata*, *Spodoptera litura*, or *S. exigua* [20,21,23]. However, these noctuid hosts require individual rearing due to cannibalism and strong larval interference under crowded conditions, resulting in high labor input, increased costs, and limited scalability [22]. In addition, prolonged maintenance on a single host species may negatively affect parasitoid performance, whereas the use of alternative or supplementary hosts has been shown to help maintain colony vigor and reproductive output in several parasitoid systems [24,25,26].

The rice moth, *Corcyra cephalonica* (Stainton) (Lepidoptera: Pyralidae), represents a potential alternative host for future rearing programs [27,28]. This cosmopolitan stored-product pest can be easily maintained on inexpensive artificial diets and reproduces continuously under controlled laboratory conditions [29,30]. *Corcyra cephalonica* has been widely used as a factitious host for mass production of biological control agents, including *Trichogramma* spp., several braconid parasitoids, and predatory insects [6,7,25,31,32]. Importantly, larvae of *C. cephalonica* are gregarious but exhibit little or no cannibalism, making them particularly suitable for high-density rearing systems [29,33].

In the present study, we conducted a preliminary laboratory evaluation of *C. cephalonica* larvae as a candidate factitious host for *M. pulchricornis*, with the northern armyworm, *M. separata* (Lepidoptera: Noctuidae), serving as a reference host. Host suitability was assessed through parasitism rate, cocooning rate, and eclosion rate under different host body lengths and parasitoid-to-host ratios. To address whether cocoon formation was followed by functional offspring performance, we further evaluated offspring quality by measuring adult body length, total egg number, and subsequent parasitism, cocooning, and eclosion performance. The aim was to generate foundational data for assessing the potential and limitations of *C. cephalonica* as a candidate host for future rearing-system optimization.

## 2. Materials and Methods

### 2.1. Insects and Rearing Conditions

A thelytokous (parthenogenetic) strain of *M. pulchricornis* was originally collected in Ningbo, Zhejiang Province, China (30.20° N, 121.03° E) in November 2021, and maintained for multiple generations in the laboratory using *M. separata* larvae as hosts in Beijing. Colonies of *C. cephalonica* and *M. separata* were reared continuously to supply host larvae for experiments.

All insect cultures were maintained in a climate chamber at 26 ± 1 °C, 60 ± 5% relative humidity (RH), and a photoperiod of 16L:8D. Unless otherwise specified, all parasitism assays were conducted under the same temperature, humidity, and photoperiod conditions.

To standardize host size across treatments, larvae were selected based on body length as a practical operational proxy for larval instar. For *C. cephalonica*, larvae measuring approximately 10, 13, and 15 mm were used, corresponding to the fifth, sixth, and seventh instars, respectively [16]. For the reference host, *M. separata* larvae of comparable body lengths were selected. Host body length was used as an operational proxy for larval instar, as this criterion is practical and commonly applied in mass-rearing systems. Only late instars were used to minimize physiological variation associated with early larval development.

To minimize variation associated with female age and physiological status, all individual parasitism assays were conducted using 5-day-old adult females, which fall within the commonly used reproductively active age range reported for *M. pulchricornis* [34].

### 2.2. Materials and Equipment

The experimental setup included a Nikonstereomicroscope SMZ800N (Nikon Corporation, Tokyo, Japan), rearing cages, plastic parasitism containers (approximately 9.3 cm in diameter × 6.8 cm in height), soft brushes, forceps, 70% ethanol for routine handling, artificial diet for host maintenance, and 10% honey solution for adult feeding. All trials were performed in controlled-environment chambers.

### 2.3. Experimental Design

#### 2.3.1. Selection of Host Instars for Assays

Pilot tests showed that parasitism on early instars (1st–4th) of *C. cephalonica* was rare, and parasitoid development was frequently unsuccessful when oviposition occurred. Therefore, only late instars of *C. cephalonica* (5th–7th) were used in subsequent experiments.

#### 2.3.2. Individual Parasitism Assays

Individual parasitism assays were designed to evaluate the effects of host body length (instar) and parasitoid-to-host ratio on parasitism, cocooning, and eclosion performance. For each replicate, a single 5-day-old female was introduced into a container with host larvae and an adequate amount of diet. Three host body length classes (approximately 10, 13, and 15 mm) were tested. For each host body length class, three parasitoid-to-host ratios were evaluated: 1:5, 1:10, and 1:15. Each treatment was replicated eight times (*n* = 8). The same experimental design was applied to *M. separata* under the same conditions. Females were allowed to parasitize hosts for 24 h, after which they were removed.

#### 2.3.3. Assessment of Parasitism, Cocooning, and Eclosion

After exposure, host larvae were monitored daily. Approximately three days after parasitism, *C. cephalonica* larvae typically darkened and died, whereas *M. separata* larvae became soft and decomposed. The number of parasitized larvae was recorded at this stage.

Successful development of *M. pulchricornis* was assessed by counting cocoons approximately seven days after parasitism, when mature larvae exited hosts and spun cocoons externally. Adult eclosion was recorded after parasitoids emerged from cocoons. Three performance metrics were calculated:

Parasitism rate (%) = (Number of parasitized larvae/Initial number of larvae) × 100(1)

Cocooning rate (%) = (Number of cocoons/Number of parasitized larvae) × 100(2)

Eclosion rate (%) = (Number of eclosed adults/Number of parasitized larvae) × 100(3)

Parasitism rate was used as an indicator of host acceptance, whereas cocooning rate and eclosion rate were used as indicators of post-parasitism developmental success.

#### 2.3.4. Assessment of Offspring Adult Quality and Subsequent Reproductive Performance

To evaluate whether host origin affected the quality and subsequent reproductive performance of *M. pulchricornis* offspring adults, adults that had developed from either *C. cephalonica* or *M. separata* were collected and assigned to two host-origin groups.

Adult body length was measured in 50 newly eclosed adults from each host-origin group under a stereomicroscope. To determine total egg number, newly eclosed female adults from each host-origin group were individually maintained under the standard rearing conditions and supplied with 10% honey solution in absorbent cotton. Each female was provided with five *M. separata* larvae for oviposition, and the host larvae were replaced every 24 h until the death of the female. Exposed host larvae were dissected 72 h after exposure, before the hatching of first-instar parasitoid larvae, and the number of parasitoid eggs was recorded. Total egg number was calculated as the cumulative number of eggs laid by each female during the assay period.

To evaluate subsequent reproductive performance, offspring adults derived from either *C. cephalonica* or *M. separata* were transferred to the *M. separata* host environment for parasitism assays. For each replicate, one parasitoid adult was provided with 5 host larvae. Host larvae were replaced every 24 h, and exposed larvae were dissected 72 h after exposure to determine egg number as described above. The assay included 10 replicates per host-origin group (*n* = 10). Parasitism rate, cocooning rate, and eclosion rate were recorded for the two host-origin groups. Adult body length was also measured to compare the body size of offspring adults derived from the two host origins.

### 2.4. Statistical Analysis

All statistical analyses were performed in R (version 4.3.2). Data are presented as mean ± SE. Parasitism rate, cocooning rate, and eclosion rate were analyzed as binomial response variables using generalized linear models (GLMs) with a logit link function. Specifically, parasitism rate was modeled using the number of parasitized and unparasitized larvae; cocooning rate was modeled using the number of cocoons and parasitized larvae that failed to produce cocoons; and eclosion rate was modeled using the number of eclosed adults and parasitized larvae that failed to produce eclosed adults.

For individual parasitism assays, the effects of host body length and parasitoid–host ratio were analyzed separately. In the host body length analysis, host species, host body length, and their interaction were included in the GLM, with parasitoid–host ratio and parasitoid age included as additional factors when applicable. In the parasitoid–host ratio analysis, host species, parasitoid–host ratio, and their interaction were included in the GLM, with host body length and parasitoid age included as additional factors when applicable. Model terms were evaluated using likelihood-ratio chi-square tests. Post hoc comparisons were performed using estimated marginal means (EMMeans) with Tukey adjustment. Analyses were based on 8 replicates per treatment for individual parasitism assays.

For the offspring adult quality and subsequent reproductive performance assay, adult body length was analyzed using a Gaussian GLM with an identity link function. Total egg number was analyzed using Poisson GLM. Subsequent parasitism rate, cocooning rate, and eclosion rate were analyzed using binomial GLMs with a logit link function. Statistical significance was determined at *p* < 0.05.

## 3. Results

### 3.1. Effect of Host Body Length on Parasitism, Cocooning, and Eclosion Rates

**Figure 1:**
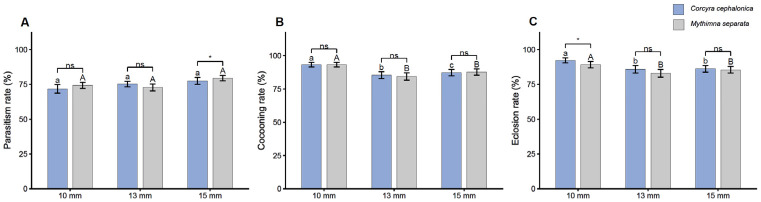
Effect of host length on parasitism rate, cocooning rate, and eclosion rate of *Meteorus pulchricornis*. (**A**) Parasitism rates of *Corcyra cephalonica* (blue bars) and *Mythimna separata* (gray bars) at different host lengths (10, 13, 15 mm) in individual parasitism experiments. (**B**) Cocooning rates of *C. cephalonica* and *M. separata* at different host lengths. (**C**) Eclosion rates of *C. cephalonica* and *M. separata* at different host lengths. Error bars represent the mean ± SE. Different lowercase letters and uppercase letters indicate significant differences in parasitism results for *C. cephalonica* and *M. separata*, respectively, at the α = 0.05 level. ‘ns’ indicates no significant difference among species under the same treatment. * *p* < 0.05.

Host body length had a limited effect on the parasitism performance of *M. pulchricornis*. In *C. cephalonica*, parasitism rates were 71.7 ± 3.0%, 75.3 ± 2.0%, and 77.5 ± 2.5% at host body lengths of 10, 13, and 15 mm, respectively. In *M. separata*, the corresponding parasitism rates were 74.3 ± 2.2%, 72.8 ± 2.4%, and 79.6 ± 1.9%, respectively (Figure 1A). The GLM detected a significant overall effect of host body length on parasitism rate (deviance = 7.53, df = 2, *p* = 0.023), whereas the effect of host species was not significant (deviance = 3.00, df = 1, *p* = 0.083). The interaction between host species and body length was also not significant (deviance = 4.14, df = 2, *p* = 0.126). Post hoc comparisons within each host species did not detect significant differences among the three host body lengths. When the two host species were compared at the same body length, parasitism rates differed significantly only at 15 mm, whereas no significant differences were detected at 10 or 13 mm.

Cocooning rate differed more clearly between host species. In *C. cephalonica*, cocooning rates were 93.2 ± 1.8%, 85.4 ± 2.6%, and 87.3 ± 2.3% at 10, 13, and 15 mm, respectively (Figure 1B). In *M. separata*, the corresponding values were 93.2 ± 1.8%, 84.3 ± 2.7%, and 87.7 ± 2.3%. The GLM showed a significant effect of host body length on cocooning rate (deviance = 63.43, df = 2, *p* < 0.001), whereas the effect of host species was not significant (deviance = 0.52, df = 1, *p* = 0.469). The interaction between host species and body length was also not significant (deviance = 0.42, df = 2, *p* = 0.810). Within *C. cephalonica*, cocooning rate differed significantly among the three body lengths. Within *M. separata*, the cocooning rate at 10 mm was significantly higher than that at 13 and 15 mm, whereas the latter two body lengths did not differ significantly.

Eclosion rate was influenced by host body length, while most pairwise comparisons between host species were not significant. In *C. cephalonica*, eclosion rates were 92.2 ± 1.7%, 85.8 ± 2.6%, and 86.1 ± 2.4% at 10, 13, and 15 mm, respectively. In *M. separata*, the corresponding rates were 89.1 ± 2.3%, 83.0 ± 2.8%, and 85.5 ± 2.4%, respectively (Figure 1C). The GLM detected significant effects of host species (deviance = 4.38, df = 1, *p* = 0.036) and host body length (deviance = 46.49, df = 2, *p* < 0.001), whereas their interaction was not significant (deviance = 1.64, df = 2, *p* = 0.441). Within both host species, eclosion rate at 10 mm was significantly higher than that at 13 and 15 mm, whereas the latter two body lengths did not differ significantly. When the two host species were compared at the same body length, a significant difference was detected only at 10 mm, while no significant differences were detected at 13 or 15 mm.

### 3.2. Effect of Parasitoid–Host Ratio on Parasitism, Cocooning, and Eclosion Rates

**Figure 2:**
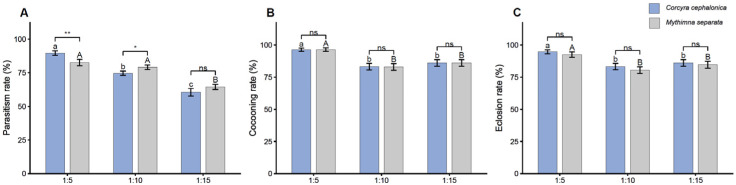
Effect of different parasitoid–host ratios on parasitism rate, cocooning rate, and eclosion rate of *Meteorus pulchricornis*. (**A**) Parasitism rates of *Corcyra cephalonica* and *Mythimna separata* at different parasitoid–host ratios (1:5, 1:10, 1:15) in individual parasitism experiments. (**B**) Cocooning rates of *C. cephalonica* and *M. separata* at different parasitoid–host ratios. (**C**) Eclosion rates at different parasitoid–host ratios. Error bars represent the mean ± SE. Different lowercase letters and uppercase letters indicate significant differences in parasitism results for *C. cephalonica* and *M. separata*, respectively, at the α = 0.05 level. ‘ns’ indicates no significant difference among species under the same treatment. * *p* < 0.05, ** *p* < 0.01.

Parasitoid–host ratio strongly affected parasitism rate. In *C. cephalonica*, parasitism rate decreased from 89.6 ± 1.7% at 1:5 to 74.6 ± 1.6% at 1:10 and 60.5 ± 2.8% at 1:15 (Figure 2A). In *M. separata*, parasitism rates were 82.5 ± 2.3%, 79.2 ± 1.7%, and 64.4 ± 1.9% at 1:5, 1:10, and 1:15, respectively. The GLM showed a significant effect of parasitoid–host ratio on parasitism rate (deviance = 182.41, df = 2, *p* < 0.001), whereas the overall effect of host species was not significant (deviance = 3.00, df = 1, *p* = 0.083). A significant interaction between host species and parasitoid–host ratio was detected (deviance = 12.77, df = 2, *p* = 0.002), indicating that the response to host density differed between the two host species. In *C. cephalonica*, parasitism rate differed significantly among all three parasitoid–host ratios. In *M. separata*, the parasitism rate at 1:15 was significantly lower than those at 1:5 and 1:10, whereas the difference between 1:5 and 1:10 was not significant. Between host species, parasitism rates differed significantly at 1:5 and 1:10, but not at 1:15.

Cocooning rate was also affected by the parasitoid–host ratio. In *C. cephalonica*, cocooning rates were 96.3 ± 1.3%, 83.1 ± 2.6%, and 86.0 ± 2.5% at ratios of 1:5, 1:10 and 1:15, respectively (Figure 2B). In *M. separata*, cocooning rates were 96.3 ± 1.4%, 82.9 ± 2.6% and 86.1 ± 2.6%, respectively. The GLM detected a significant effect of parasitoid–host ratio on cocooning rate (deviance = 71.11, df = 2, *p* < 0.001), whereas the effect of host species was not significant (deviance = 0.52, df = 1, *p* = 0.469). The interaction between host species and parasitoid–host ratio was also not significant (deviance = 0.16, df = 2, *p* = 0.925). Within *C. cephalonica*, the cocooning rate at 1:5 was significantly higher than those at 1:10 and 1:15, whereas the latter two ratios did not differ significantly. Within *M. separata*, cocooning rate was significantly higher at 1:5 than at 1:10 or 1:15, while the latter two ratios did not differ significantly. No significant differences between host species were detected at any parasitoid–host ratio.

Eclosion rate was affected by parasitoid–host ratio, while pairwise comparisons between host species were not significant at any parasitoid–host ratio. In *C. cephalonica*, eclosion rates were 94.6 ± 1.5%, 83.2 ± 2.5%, and 86.0 ± 2.6% at 1:5, 1:10, and 1:15, respectively. In *M. separata*, the corresponding values were 92.5 ± 2.1%, 80.5 ± 2.6%, and 84.7 ± 2.6%, respectively (Figure 2C). The GLM detected significant effects of host species (deviance = 4.38, df = 1, *p* = 0.036) and parasitoid–host ratio (deviance = 49.03, df = 2, *p* < 0.001), whereas their interaction was not significant (deviance = 0.34, df = 2, *p* = 0.844). Within both host species, eclosion rate at 1:5 was significantly higher than that at 1:10 and 1:15, whereas the latter two ratios did not differ significantly. When the two host species were compared at the same parasitoid–host ratio, no significant differences were detected at 1:5, 1:10, or 1:15.

### 3.3. Quality and Subsequent Reproductive Performance of Offspring Adults from Different Host Origins

To further evaluate the quality and reproductive performance of offspring adults produced from different host origins, we compared adult body length and total egg number of *M. pulchricornis* adults derived from *C. cephalonica* and *M. separata*. We then assessed the subsequent parasitism, cocooning, and eclosion rates produced by these offspring adults when exposed to host larvae under standardized conditions.

**Figure 3:**
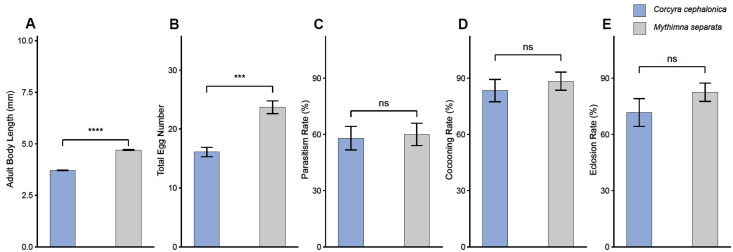
Quality and subsequent reproductive performance of *Meteorus pulchricornis* offspring adults derived from different host origins. (**A**) Adult body length of *M. pulchricornis* adults derived from *Corcyra cephalonica* and *Mythimna separata*. (**B**) Total egg number of females derived from the two host origins. (**C**) Subsequent parasitism rate produced by offspring adults from the two host origins. (**D**) Subsequent cocooning rate produced by offspring adults from the two host origins. (**E**) Subsequent eclosion rate produced by offspring adults from the two host origins. Blue bars indicate the *C. cephalonica* host-origin group, and gray bars indicate the *M. separata* host-origin group. Error bars represent the mean ± SE. ‘ns’ indicates no significant difference among species under the same treatment. *** *p* < 0.001, **** *p* < 0.0001.

Adult body length differed significantly between the two host-origin groups. Adults derived from *C. cephalonica* were smaller than those derived from *M. separata*, with mean body lengths of 3.71 ± 0.02 mm and 4.70 ± 0.02 mm, respectively (Figure 3A; Gaussian GLM: F = 1455.60, df = 1, 98, *p* < 0.001). Total egg number was also significantly lower in adults derived from *C. cephalonica* than in those derived from *M. separata* (16.1 ± 0.8 vs. 23.7 ± 1.1 eggs; Figure 3B; Poisson GLM: deviance = 14.60, df = 1, *p* < 0.001).

In contrast, subsequent reproductive performance did not differ significantly between the two host-origin groups. Subsequent parasitism rates were 58.0 ± 6.3% for *C. cephalonica*-derived adults and 60.0 ± 6.0% for *M. separata*-derived adults (Figure 3C; binomial GLM: deviance = 0.17, df = 1, *p* = 0.683). Subsequent cocooning rates were 83.4 ± 5.9% and 88.4 ± 4.8%, respectively (Figure 3D; binomial GLM: deviance = 0.22, df = 1, *p* = 0.638). Subsequent eclosion rates were 71.7 ± 7.4% and 82.5 ± 4.9%, respectively (Figure 3E; binomial GLM: deviance = 1.06, df = 1, *p* = 0.303).

Overall, offspring adults derived from *C. cephalonica* were smaller and had fewer eggs than those derived from *M. separata*, but their subsequent parasitism, cocooning and eclosion rates were not significantly different. These results indicate that *C. cephalonica* can produce functional *M. pulchricornis* offspring adults, although some adult-quality traits were reduced compared with those produced from *M. separata*.

## 4. Discussion

This study evaluated *C. cephalonica* as a candidate factitious host for *M. pulchricornis* under laboratory conditions, using *M. separata* as a reference host. We focused on host body length, parasitoid-to-host ratio, and the quality and subsequent reproductive performance of offspring adults derived from different host origins. *Corcyra cephalonica* supported parasitism, cocoon formation, and adult eclosion with cocooning performance generally comparable to that of the reference host *M. separata*. The offspring-quality assessment further showed that adults derived from *C. cephalonica* were smaller and had fewer eggs than those derived from *M. separata*, but their subsequent parasitism, cocooning, and eclosion rates did not differ significantly from those of adults derived from the reference host. Together, these results suggest that *C. cephalonica* can support the development of functional *M. pulchricornis* adults, although it may not be fully equivalent to *M. separata* in terms of adult size and reproductive potential.

### 4.1. Host Size as an Operational Criterion

In applied rearing systems, host size is often more practical than precise instar determination. Within the tested range of late instars (fifth to seventh, corresponding to body lengths of approximately 10–15 mm), parasitism rate, cocooning and eclosion rates were not significantly different among host size classes. This indicates that *M. pulchricornis* can accept and parasitize *C. cephalonica* larvae across this late-instar size window, but post-parasitism developmental success may still be influenced by host body length.

Although the parasitism rate did not differ significantly among the tested body length classes, cocooning and eclosion rates were generally higher in the 10 mm group than in the larger body length classes. Similar patterns have been reported in other koinobiont endoparasitoids, where late instars generally provide sufficient nutritional resources but do not impose strong size-dependent constraints once a minimum threshold is exceeded [12,35,36,37]. For *M. pulchricornis*, host age or size effects have been shown to vary among host species and experimental systems [35,37,38,39], suggesting that host identity and rearing context can modulate size-related responses. Cocooning rates did not differ significantly between *C. cephalonica* and *M. separata* at any matched body length, whereas interspecific differences in eclosion rate were limited to specific body length comparisons. This suggests that host body length influenced post-parasitism developmental success, but the overall differences between the candidate host and the reference host were limited under matched body length conditions. However, the lack of significant differences in parasitism rate within *C. cephalonica* does not necessarily imply equivalent developmental performance or offspring quality across this size range. Overall, late instars within the tested range (10–15 mm) are broadly suitable from the perspective of host acceptance, immature development, and adult eclosion.

### 4.2. Parasitoid–Host Ratio and Density Effects

Parasitoid–host ratio strongly influenced performance on both host species. The highest parasitism, cocooning, and eclosion rates generally occurred at a ratio of 1:5, with reduced performance at higher host densities. Eclosion rate also varied among parasitoid–host ratios, indicating that host density affected not only host acceptance and cocoon formation but also overall post-parasitism development to adult eclosion. Density-dependent effects on parasitoid efficiency are well documented and are often attributed to a combination of reduced encounter efficiency, increased interference, and superparasitism under crowded conditions [40,41,42].

Although the detailed parasitism response differed between *C. cephalonica* and *M. separata*, most pairwise comparisons of cocooning and eclosion rates between host species were not significant under the same parasitoid–host ratios. This suggests that the main variation in developmental performance was associated more with parasitoid–host ratio than with host species under the tested conditions. From an applied perspective, the 1:5 ratio appears to provide the most reliable performance across parasitism, cocooning, and eclosion, whereas higher host densities may reduce proportional developmental success.

### 4.3. Offspring Adult Quality and Subsequent Reproductive Performance

Offspring adult quality is an important component of host suitability because successful cocoon formation does not necessarily indicate that the resulting adults are fully functional. In the present study, adults derived from *C. cephalonica* were significantly smaller and contained fewer eggs than those derived from *M. separata*. These results indicate that host origin affected adult body size and reproductive potential, suggesting that *C. cephalonica* may provide a less optimal nutritional or physiological environment for immature parasitoid development than the reference host.

Despite these differences, adults derived from *C. cephalonica* showed subsequent parasitism, cocooning, and eclosion rates comparable to those derived from *M. separata* under standardized assay conditions. This indicates that *C. cephalonica* can produce functional *M. pulchricornis* adults capable of parasitizing hosts and supporting post-parasitism development to adult eclosion. Therefore, *C. cephalonica* shows potential as a candidate factitious host, but the reduced adult body length and egg number suggest that it should not yet be considered fully equivalent to *M. separata*. Further optimization should aim to improve both rearing efficiency and adult-quality traits.

### 4.4. Limitations and Future Directions

Several limitations should be considered when interpreting these results. First, although adult body length, egg number, and subsequent reproductive performance were evaluated, additional fitness-related traits, such as developmental time, lifetime fecundity, longevity, multi-generational performance, and post-release performance, were not assessed and are necessary to fully evaluate host quality [21,43,44,45]. Second, all experiments were conducted under controlled laboratory conditions. Consequently, the stability and ecological relevance of the observed patterns under variable field conditions (e.g., fluctuating temperature, humidity, or host availability) remain unknown [2].

Future work should aim to (i) evaluate multi-generational effects and potential fitness changes, and (ii) test whether short, standardized pre-parasitism conditioning can be incorporated into routine rearing procedures [46]. Given evidence that periodic host switching can mitigate fitness declines in parasitoid colonies, the use of *C. cephalonica* as part of a flexible host-rearing strategy also merits further investigation [26,47,48,49].

## Data Availability

The data presented in this study are available on request from the corresponding author.

## References

[1] Zhou W., Arcot Y., Medina R.F., Bernal J., Cisneros-Zevallos L., Akbulut M.E.S. (2024). Integrated pest management: An update on the sustainability approach to crop protection. ACS Omega.

[2] Heimpel G.E., Mills N.J. (2017). Biological Control: Ecology and Applications.

[3] Li B., Duan Y., Du Z., Wang X., Liu S., Feng Z., Tian L., Song F., Yang H., Cai W. (2024). Natural selection and genetic diversity maintenance in a parasitic wasp during continuous biological control application. Nat. Commun..

[4] Burke G.R., Sharanowski B.J. (2024). Parasitoid wasps. Curr. Biol..

[5] Lü X., Qiu R., He X., Li J. (2024). Evaluation of key factors for mass rearing the egg parasitoid Telenomus remus Nixon (Hymenoptera: Scelionidae). CABI Agric. Biosci..

[6] Grenier S., De Clercq P., van Lenteren J.C. (2003). Comparison of artificially vs. naturally reared natural enemies and their potential for use in biological control. Quality Control and Production of Biological Control Agents: Theory and Testing Procedures.

[7] van Lenteren J.C. (2012). The state of commercial augmentative biological control: Plenty of natural enemies, but a frustrating lack of uptake. BioControl.

[8] Shen Z., Huang M., Tariq H., Wei J., Kamfwa K., Bueno A.d.F., Tang L., Zang L. (2025). Economics trumps biology: *Corcyra cephalonica* as the optimal factitious host for cost-effective mass production of *Chelonus bifoveolatus*, a key parasitoid of *Spodoptera frugiperda*. Pest Manag. Sci..

[9] Maeto K. (1990). Phylogenetic relationships and host associations of the subfamily Meteorinae Cresson (Hymenoptera: Braconidae). Jpn. J. Entomol..

[10] Berry J.A., Walker G.P. (2004). *Meteorus pulchricornis* (Wesmael) (Hymenoptera: Braconidae: Euphorinae): An exotic polyphagous parasitoid in New Zealand. N. Z. J. Zool..

[11] Suzuki M., Tanaka T. (2007). Development of *Meteorus pulchricornis* and regulation of its noctuid host, *Pseudaletia separata*. J. Insect Physiol..

[12] Maeto K. (2018). Polyphagous koinobiosis: The biology and biocontrol potential of a braconid endoparasitoid of exophytic caterpillars. Appl. Entomol. Zool..

[13] Chen Y., Wang P., Shu X., Wang Z., Chen X. (2023). Morphology and ultrastructure of the female reproductive apparatus of an asexual strain of the endoparasitoid *Meteorus pulchricornis* (Wesmael) (Hymenoptera: Braconidae). Biology.

[14] Fujie S., Wachi N., Umemoto H., Maeto K. (2019). Mitochondrial DNA diversity and geographical distribution of sexual and asexual strains of the braconid parasitoid *Meteorus pulchricornis*. Entomol. Exp. Appl..

[15] Tsutsui Y., Maeto K., Hamaguchi K., Isaki Y., Takami Y., Naito T., Miura K. (2014). Apomictic parthenogenesis in a parasitoid wasp *Meteorus pulchricornis*, uncommon in the haplodiploid order Hymenoptera. Bull. Entomol. Res..

[16] Li G.J., Jiang F.L., Guo J.J. (1982). Morphology and habits of the rice moth *Corcyra cephalonica*. Kun Chong Zhi Shi.

[17] Guo H.-F., Fang J.-C., Zhong W.-F., Liu B.-S. (2013). Interactions between *Meteorus pulchricornis* and *Spodoptera exigua* multiple nucleopolyhedrovirus. J. Insect Sci..

[18] Zhou J.C., Meng L., Li B.P. (2017). Defensive behaviors of the oriental armyworm *Mythimna separata* in response to different parasitoid species (Hymenoptera: Braconidae). PeerJ.

[19] Nakano S., Gau J.J., Maeto K. (2018). Host suitability of the Mediterranean flour moth for rearing *Meteorus pulchricornis* (Hymenoptera: Braconidae), a polyphagous endoparasitoid of pest lepidopteran larvae. Appl. Entomol. Zool..

[20] Liu Y.H., Li B.P. (2008). Effects of *Helicoverpa armigera* (Noctuidae, Lepidoptera) host stages on some developmental parameters of the uniparental endoparasitoid *Meteorus pulchricornis* (Braconidae, Hymenoptera). Bull. Entomol. Res..

[21] Wang H.P., Meng L., Li B.P. (2008). Effects of feeding frequency and sugar concentrations on lifetime reproductive success of *Meteorus pulchricornis* (Hymenoptera: Braconidae). Biol. Control.

[22] De Clercq P. (2024). Plants in the rearing of arthropod predators and parasitoids: Benefits, constraints, and alternatives. Curr. Opin. Insect Sci..

[23] Liu Z., Huang L., Meng L. (2010). Host selection and offspring performance of *Meteorus pulchricornis* on different host species. Sheng Tai Xue Bao.

[24] Chen W., Li Y., Wang M., Mao J., Zhang L. (2021). Evaluating the potential of using *Spodoptera litura* eggs for mass-rearing *Telenomus remus*, a Promising Egg Parasitoid of *Spodoptera frugiperda*. Insects.

[25] Shen Z., Chen Y.-M., Huang M., Tariq H., Tang L.-D., Wei J., Bai Q.-Y., Kamfwa K., Ramírez-Romero R., Monticelli L.S. (2025). Mass rearing of *Chelonus* parasitoids with the alternative host *Corcyra cephalonica*: A promising biocontrol option against fall armyworm *Spodoptera frugiperda*. Pest Manag. Sci..

[26] DBastiani E., Princepe D., Marquitti F.M.D., Boeger W.A., Campião K.M., Araujo S.B.L. (2023). Effect of host-switching on the ecological and evolutionary patterns of parasites. Syst. Biol..

[27] Manjunath T.M. (2023). Rice moth, *Corcyra cephalonica* (Lepidoptera, Pyralidae)—A boon for biocontrol as a factitious host for mass production of parasitoids and predators. J. Biol. Control.

[28] Queiroz A.P., de Freitas Bueno A., Pomari-Fernandes A., Grande M.L.M., Bortolotto O.C., da Silva D.M. (2017). Quality control of *Telenomus remus* (Hymenoptera: Platygastridae) reared on the factitious host *Corcyra cephalonica* (Lepidoptera: Pyralidae) for successive generations. Bull. Entomol. Res..

[29] Bernardi E.B., Haddad M.L., Parra J.R. (2000). Comparison of artificial diets for rearing *Corcyra cephalonica* (Stainton, 1865) (Lep., Pyralidae) for *Trichogramma* mass production. Rev. Bras. Biol..

[30] Vincent A., Singh D., Mathew I.L. (2021). *Corcyra cephalonica*: A serious pest of stored products or a factitious host of biocontrol agents? *J*. Stored Prod. Res..

[31] Gowda B.G., Pandi G.P., Ullah F., Patil N.B., Sahu M., Adak T., Pokhare S., Yadav M.K., Mahendiran A., Mittapelly P. (2021). Performance of *Trichogramma japonicum* under field conditions as a function of the factitious host species used for mass rearing. PLoS ONE.

[32] Migiro L.N., Gitonga L.M., Sithanantham S. (2008). Evaluation of *Ephestia kuehniella* and *Corcyra cephalonica* as hosts for mass rearing *Trichogramma* species nr. Mwanzai and trichogrammatoidea species nr. *Lutea*. J. Agric. Sci. Technol..

[33] Nath A., Gadratagi B.G., Maurya R.P., Ullah F., Patil N.B., Adak T., Govindharaj G.P.P., Ray A., Mahendiran A., Desneux N. (2023). Sublethal phosphine fumigation induces transgenerational hormesis in a factitious host, Corcyra cephalonica. Pest Manag. Sci..

[34] Liu Y.-H., Li B.-P. (2006). Developmental interactions between *Spodoptera exigua* (Noctuidae: Lepidoptera) and its uniparental endoparasitoid, *Meteorus pulchricornis* (Braconidae: Hymenoptera). Biol. Control.

[35] Harvey J.A., Molina A.C., Bezemer T.M., Malcicka M. (2015). Convergent development of a parasitoid wasp on three host species with differing mass and growth potential. Entomol. Exp. Appl..

[36] Harvey J.A., Sano T., Tanaka T. (2010). Differential host growth regulation by the solitary endoparasitoid, *Meteorus pulchricornis* in two hosts of greatly differing mass. J. Insect Physiol..

[37] Liu Y.H., Li B.P., Xu Z.H. (2013). Effect of host instar and temperature on fitness-related traits in the solitary endoparasitoid, *Meteorus pulchricornis*. Phytoparasitica.

[38] Sharma A., Sharma P.L., Verma S.C., Chandel R.S., Chandel V.G.S., Sharma D., Semwal A., Sharma N. (2025). The demographic and life table studies of *Corcyra cephalonica* on different food grains and their impact on population growth parameters and life history traits of indigenous *Trichogramma chilonis* and *T. achaeae*. Crop Prot..

[39] Fuester R.W., Taylor P.B., Peng H., Swan K. (1993). Laboratory biology of a uniparental strain of *Meteorus pulchricornis* (Hymenoptera: Braconidae), An Exotic Larval Parasite of the Gypsy Moth (Lepidoptera: Lymantriidae). Ann. Entomol. Soc. Am..

[40] Vos M., Hemerik L. (2003). Linking foraging behavior to lifetime reproductive success for an insect parasitoid: Adaptation to host distributions. Behav. Ecol..

[41] Wajnberg É., Bernstein C., van Alphen J. (2008). Behavioral Ecology of Insect Parasitoids.

[42] Clemente G., Toledo J., Pérez-Lachaud G., Valle-Mora J.F., Liedo P., Montoya P. (2024). Functional response and mutual interference in the parasitoid *Coptera haywardi* (Oglobin) (Hymenoptera: Diapriidae) attacking *Anastrepha ludens* (Loew) (Diptera: Tephritidae) pupaet. Bull. Entomol. Res..

[43] van Lenteren J.C. (2003). Quality Control and Production of Biological Control Agents: Theory and Testing Procedures.

[44] Godfray H.C.J. (1994). Parasitoids: Behavioral and Evolutionary Ecology.

[45] Harvey J.A. (2005). Factors affecting the evolution of development strategies in parasitoid wasps: The importance of functional constraints and incorporating complexity. Entomol. Exp. Appl..

[46] Papaj D.R., Prokopy R.J. (1989). Ecological and evolutionary aspects of learning in phytophagous insects. Annu. Rev. Entomol..

[47] Mácová A., Hoblíková A., Hypša V., Stanko M., Martinů J., Kvičerová J. (2018). Mysteries of host switching: Diversification and host specificity in rodent–coccidia associations. Mol. Phylogenet. Evol..

[48] Nunney L., van Lenteren J.C. (2003). Managing captive populations for release: A population-genetic perspective. Quality Control and Production of Biological Control Agents: Theory and Testing Procedures.

[49] Mills N.J., Kean J.M. (2010). Behavioral studies, molecular approaches, and modeling: Methodological contributions to biological control success. Biol. Control.

